# Neutralizing and enhancing monoclonal antibodies in SARS-CoV-2 convalescent patients: lessons from early variant infection and impact on shaping emerging variants

**DOI:** 10.1080/22221751.2024.2307510

**Published:** 2024-01-19

**Authors:** Frédéric Coutant, Franck Touret, Jean-Jacques Pin, Marina Alonzo, Cécile Baronti, Sandie Munier, Mikaël Attia, Xavier de Lamballerie, Tristan Ferry, Pierre Miossec

**Affiliations:** a Immunogenomics and Inflammation Research Team, University of Lyon, Edouard Herriot Hospital, Lyon, France; b Immunology Department, Lyon-Sud Hospital, Hospices Civils of Lyon, Pierre-Bénite, France; c Unité des Virus Émergents (UVE: Aix-Marseille University - IRD 190 - Inserm 1207), Marseille, France; d Eurobio Scientific/Dendritics – Edouard Herriot Hospital, Lyon, France; e Institut Pasteur, Université Paris Cité, CNRS UMR3569, Molecular Genetics of RNA Viruses Unit, Paris, France; f Department of Infectious and Tropical Diseases, Hospices Civils of Lyon - Croix-Rousse Hospital, Lyon, France; g CIRI, Inserm U1111, CNRS, UMR5308, ENS Lyon, Université Claude Bernard Lyon I, Lyon, France; h Department of Immunology and Rheumatology, Edouard Herriot Hospital, Lyon, France

**Keywords:** COVID-19, B cell repertoire, antibody-dependent enhancement, human monoclonal antibody, emerging variants

## Abstract

Serological studies of COVID-19 convalescent patients have identified polyclonal lineage-specific and cross-reactive antibodies (Abs), with varying effector functions against virus variants. Individual specificities of anti-SARS-CoV-2 Abs and their impact on infectivity by other variants have been little investigated to date. Here, we dissected at a monoclonal level neutralizing and enhancing Abs elicited by early variants and how they affect infectivity of emerging variants. B cells from 13 convalescent patients originally infected by D614G or Alpha variants were immortalized to isolate 445 naturally-produced anti-SARS-CoV-2 Abs. Monoclonal antibodies (mAbs) were tested for their abilities to impact the cytopathic effect of D614G, Delta, and Omicron (BA.1) variants. Ninety-eight exhibited robust neutralization against at least one of the three variant types, while 309 showed minimal or no impact on infectivity. Thirty-eight mAbs enhanced infectivity of SARS-CoV-2. Infection with D614G/Alpha variants generated variant-specific (65 neutralizing Abs, 35 enhancing Abs) and cross-reactive (18 neutralizing Abs, 3 enhancing Abs) mAbs. Interestingly, among the neutralizing mAbs with cross-reactivity restricted to two of the three variants tested, none demonstrated specific neutralization of the Delta and Omicron variants. In contrast, cross-reactive mAbs enhancing infectivity (*n* = 3) were found exclusively specific to Delta and Omicron variants. Notably, two mAbs that amplified *in vitro* the cytopathic effect of the Delta variant also exhibited neutralization against Omicron. These findings shed light on functional diversity of cross-reactive Abs generated during SARS-CoV-2 infection and illustrate how the balance between neutralizing and enhancing Abs facilitate variant emergence.

## Introduction

During the course of the COVID-19 pandemic, several variants of concern of the severe acute respiratory syndrome coronavirus 2 (SARS-CoV-2) have emerged and spread globally, including Alpha (also known as lineage B.1.1.7), Delta (B.1.617.2), and Omicron (B.1.1.529). The antibody (Ab) response following SARS-CoV-2 infection has been extensively examined at the polyclonal level using immune sera, as well as the dynamics of variant escape to neutralizing Abs [1–4]. Most of the studies on the humoral immune response triggered by SARS-CoV-2 has focused on Abs that target the spike protein, particularly the receptor binding domain and the N-terminal domain, which are the two domains where mutations accumulate, leading to neutralization escape by emerging variants [5]. However, SARS-CoV-2 infection elicits a broad and complex polyclonal immune response, including not only neutralizing Abs but also facilitating Abs, as well as Abs that target autoantigens [6–9]. Serological studies provide only a glimpse of the intricate interplay of individual Abs effects induced by SARS-CoV-2 infection. In addition to the well-studied neutralizing Abs, non-neutralizing Abs can also contribute to the clearance of the virus through Fc effector cytotoxic functions, while others, referred to as enhancing Abs, can facilitate the infectivity of the virus, a mechanism known as Ab-dependent enhancement [10–13]. Facilitating antibodies targeting the N-terminal domain (NTD) or the receptor binding domain (RBD) of the spike protein have been isolated and characterized *in vitro* for SARS-CoV-2 [6,7]. Interestingly, when evaluated *in vivo*, in mice or non-human primates, they exhibited a protective effect [7]. Additionally, SARS-CoV-2 infection induces the generation of multiple Abs with functions that remain poorly understood. These Abs target non-canonical antigens, *i.e.* antigens other than the spike and nucleocapsid proteins or their subdomains, expanding the complexity of the antiviral immune response [14].

Serological studies have significantly broadened our understanding of the functional and specificity diversity of Abs produced in response to SARS-CoV-2 infection at the polyclonal level. However, our knowledge regarding the anti-SARS-CoV-2 Ab response at the monoclonal level in convalescent patients infected with early variants, as well as the functional properties of these induced monoclonal Abs (mAbs) in relation to emerging variants, remains limited. To address this critical gap, we isolated a panel of 445 naturally produced anti-SARS-CoV-2 mAbs from the B cells of 13 convalescent patients initially infected with either the D614G or Alpha variants. Subsequently, we assessed their functional properties against emerging variants. We have identified a delicate balance between neutralizing and enhancing Abs, each displaying varying degrees of specificity towards emerging variants. Notably, we point out the existence of cross-reactive anti-SARS-CoV-2 Abs exhibiting divergent functionality when confronted with SARS-CoV-2 variants.

## Materials and methods

### Sample donors

Samples were collected from individuals who had recovered from SARS-CoV-2 infection, vaccinated or not. The study protocols were approved by the institutional review board for ethics (Ethics Committee of the Hospitals of Lyon for the Protection of People; IDRCB 2020-A01038-31). Prior to participating in the study, all participants provided written informed consent. The study is registered on ClinicalTrials.gov (NCT04354766).

### Cell lines

VeroE6/TMPRSS2 cells (ID 100978) were obtained from CFAR and were grown in minimal essential medium (Life Technologies). The culture medium was supplemented with 7.5% heat-inactivated fetal calf serum (FCS; Life Technologies), 1% penicillin/streptomycin (PS, 5000U.mL−1 and 5000 µg.mL−1 respectively; Life Technologies), 1% non-essential amino acids (Life Technologies) and 1 mg/ml of G-418 (Life Technologies). The cells were maintained at 37°C with 5% CO2.

### Virus strain

The SARS-CoV-2 strain BavPat1 was obtained from Pr. C. Drosten through EVA GLOBAL (https://www.european-virus-archive.com/). This strain belongs to the B.1 lineage and contains the D614G mutation.

The SARS-CoV-2 Delta variant (B.1.617.2 strain, hCoV-19/France/PAC-0610/2021) was isolated in May 2021 in Marseille, France. This variant belongs to the AY.71 lineage and its full genome sequence has been deposited on GISAID: EPI_ISL_2838050. The strain is available through EVA GLOBAL (www.european-virus-archive.com, ref: 001 V-04282).

The SARS-CoV-2 Omicron BA.1 (B.1.1.529 strain hCoV-19/France/PAC-1514/2021) was isolated on the 1st of December 2021 in Marseille, France. This variant belongs to the BA.1.17 lineage and its full genome sequence has been deposited on GISAID: EPI_ISL_7899754. This strain is available through EVA GLOBAL (www.european-virus-archive.com, ref: 001 V-04436).

### Generation of immortalized B cell clones

Stable Ab-secreting cells were produced following a previously described protocol [15]. Briefly, cells were generated using an optimized technology involving CD40/CD40 ligand stimulation and Epstein–Barr virus transformation (Human Blood B Booster kit DDX-HUBBB-Eurobio Scientific/Dendritics, Les Ulis, France). Purified peripheral blood mononuclear cells from donors were suspended in a booster reagent medium (Eurobio Scientific/Dendritics, Les Ulis, France) and cocultured for 8 days at 37 °C with FcgRII/CDw32 cells (cell line L4, Human Blood B Booster kit, Eurobio Scientific/Dendritics, Les Ulis, France). These FcgRII/CDw32 cells had been pre-incubated with 0.5 µg/mL of anti-CD40 Ab (Mab89, Eurobio Scientific/Dendritics, Les Ulis, France), and 1 µg/mL of monoclonal B cell stimulating Ab (clones 107B5 and 110F7, Eurobio Scientific/Dendritics, Les Ulis, France), along with EBV suspension (Human Blood B Booster kit, Eurobio Scientific/Dendritics, Les Ulis, France). On day 8, the culture plates were duplicated and supplemented with 1 × 104 cells/well of lyophilized fibroblastic L6 cells that stably express the CD40 ligand (Human Blood B Booster kit, Eurobio Scientific/Dendritics, Les Ulis, France), 1 µg/mL of 107B5 and 110F7 Ab, and EBV suspension. By day 21 of culture, supernatants were screened for the presence of anti-SARS-CoV-2 Ab. Positive cultures were subjected to cloning procedures until a monoclonal stage was achieved.

### Indirect immunofluorescence assay

B cell clone supernatants were screened using an indirect immunofluorescence staining method on SARS-CoV-2–infected BGM cells or Vero cells that had been fixed with ice-cold acetone (95%). Following incubation with clone supernatants (30 min at 37 °C), the cells were washed with PBS and then further incubated with FITC-conjugated anti-human IgG,A,M Ab (Abliance, Compiègne, France) at 1:300 dilution. Cell imaging was performed using an Axioplan 2 imaging microscope (Zeiss, Munich, Germany).

### Quantification of the cytopathic effect

The assay was performed as previously described [16]. Briefly, 5 × 104 VeroE6 TMRPSS2 cells were seeded in 96 well plates in 100 µL assay medium (containing 2.5% FCS) one day before infection. On the following day, clone supernatants were added to the cells (25 µL/well, resulting in a final mAb concentration range of 500 ng/mL to 2 µg/mL, in 2.5% FCS-containing medium). Six wells served as virus control and were supplemented with 25 µL medium (positive controls), while eight wells were designated as cell controls and were supplemented with 50 µL of medium (negative controls). Two internal well controls for viral inhibition were included, involving the addition of 10 µM Remdesivir (BLDpharm, Shangai, China) to the infected cell culture. After 15 min of incubation, 25 µL of a virus mix diluted in medium containing 2.5% FCS was added to the wells at a MOI 0.002. Three days after infection, the cell culture supernatant were removed, and CellTiter-Blue reagent (PROMEGA, Fitchburg, USA) was added following the manufacturer’s instructions. The plates were incubated for 2 h before recording fluorescence (560/590 nm) using a Tecan Infinite 200Pro machine (TECAN, Zurich, Switzerland). The Inhibition Index was calculated as follows: the cell viability for clone supernatants, virus control, and viral inhibition control (Remdesivir) were determined by dividing the OD590 nm value by the mean OD590 nm of negative controls. Mean cell viabilities for virus control and viral inhibition control were then calculated. Subsequently, all cell viability values were normalized by subtracting the mean virus control cell viability. Finally, the Inhibition Index was derived as follows: Inhibition Index = normalized cell viability of the clone supernatant divided by the normalized cell viability of the viral inhibition control in the same 96-well plate.

## Results

### Functional evaluation at the monoclonal level of anti-SARS-CoV-2 antibodies following infection

To investigate the functional diversity of the Ab response induced by SARS-CoV-2 infection at the monoclonal level, we isolated a panel of 445 anti-SARS-CoV-2 Abs from 13 convalescent patients who had been infected by either the D614G or Alpha variants, with or without prior vaccination (Table 1). We employed an optimized two-step strategy to generate stable antiviral B cell clones from circulating B cells of convalescent patients. Initially, a transient proliferation/differentiation programme was induced in B cells using an optimized CD40/CD40 ligand stimulation protocol, followed by the establishment of stable B cell clones by infecting cells with Epstein–Barr virus [15,17]. This approach yielded a total of 766,500 B cell clones, encompassing a broad spectrum of B cell clones representing the global B cell repertoire. To identify virus-specific clones, we screened the B cell clone supernatants on fixed SARS-CoV-2-infected cells by indirect immunofluorescence, thereby avoiding the bias inherent to a screening process based on a recombinant viral antigen. We selected positive supernatants indicative of the presence of anti-SARS-CoV-2 Abs and cultured polyclonal B cells until they reached a monoclonal stage. Subsequently, we evaluated 445 clone supernatants containing naturally produced anti-SARS-CoV-2 Abs for their ability to protect VeroE6/ TMPRSS2 cells from the cytopathic effect induced by SARS-CoV-2 infection, following a standardized screening assay [16,18]. Each clone supernatant was individually assessed for its impact on the infectivity of the prototype D614G variant, as well as Delta and first Omicron variants (BA.1). The mAbs were categorized as either strongly neutralizing, partially/non-neutralizing, or enhancing, based on their ability to provide robust protection (inhibition index > 0.9), partial/limited protection, or exacerbation of the of the viral cytopathic effect (inhibition index < 0).

**Table 1.** Demographic and clinical characteristics of study participants.

	Gender	Age	Severity of the disease	Days from symptoms onset to sampling	Initial SARS-CoV-2 variant	Vaccination status
**P1**	F	22	Mild	150	Alpha	2 doses (Pfizer)
**P2**	M	72	Severe	26	D614G	Unvaccinated
**P3**	M	54	Severe	24	Alpha	Unvaccinated
**P4**	F	59	Mild	62	Alpha	1 dose (Pfizer)
**P5**	M	91	Severe	17	D614G	Unvaccinated
**P6**	F	60	Mild	27	D614G	Unvaccinated
**P7**	M	27	Mild	30	D614G	Unvaccinated
**P8**	M	27	Mild	39	D614G	Unvaccinated
**P9**	F	84	Severe	38	Alpha	Unvaccinated
**P10**	M	48	Mild	38	D614G	Unvaccinated
**P11**	M	56	Mild	33	Alpha	Unvaccinated
**P12**	M	31	Mild	29	Alpha	Unvaccinated
**P13**	F	50	Severe	11	Alpha	Unvaccinated

**Figure 1.**
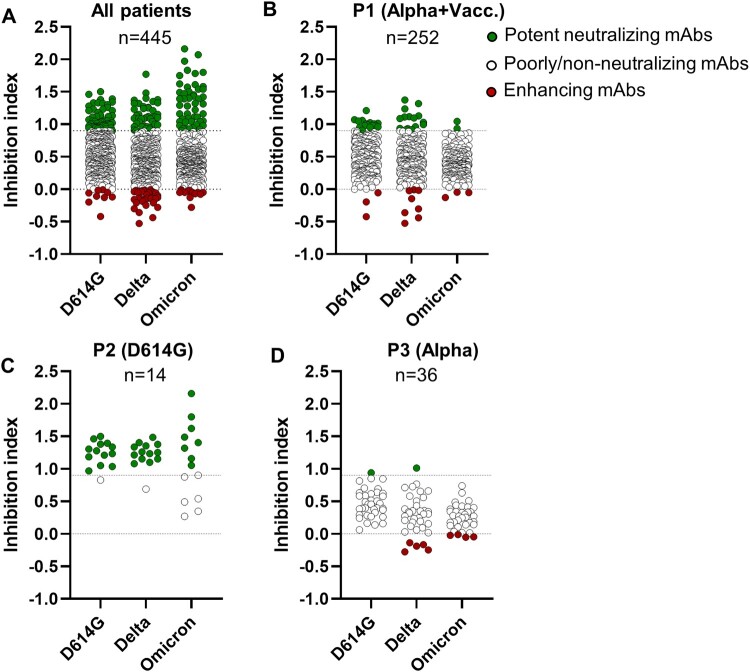
Identification of human monoclonal anti-SARS-CoV-2 antibodies with neutralizing and enhancing properties. (A) The capacity of 445 B cell clone supernatants to affect the cytopathic effect induced by SARS-CoV-2 variants was assessed by measuring cell viability three days after cell infection (MOI of 0.002). The Inhibition Index was determined by dividing the normalized cell viability achieved using clone supernatants by the normalized cell viability measured with the viral inhibition control (remdesivir). (B-D) Cytopathic effect of SARS-CoV-2 variants following incubation with clone supernatants from convalescent patients infected with the Alpha variant, either vaccinated (patient 1) or unvaccinated (patient 3), or infected with the D614G variant (patient 2).

**Figure 2.**
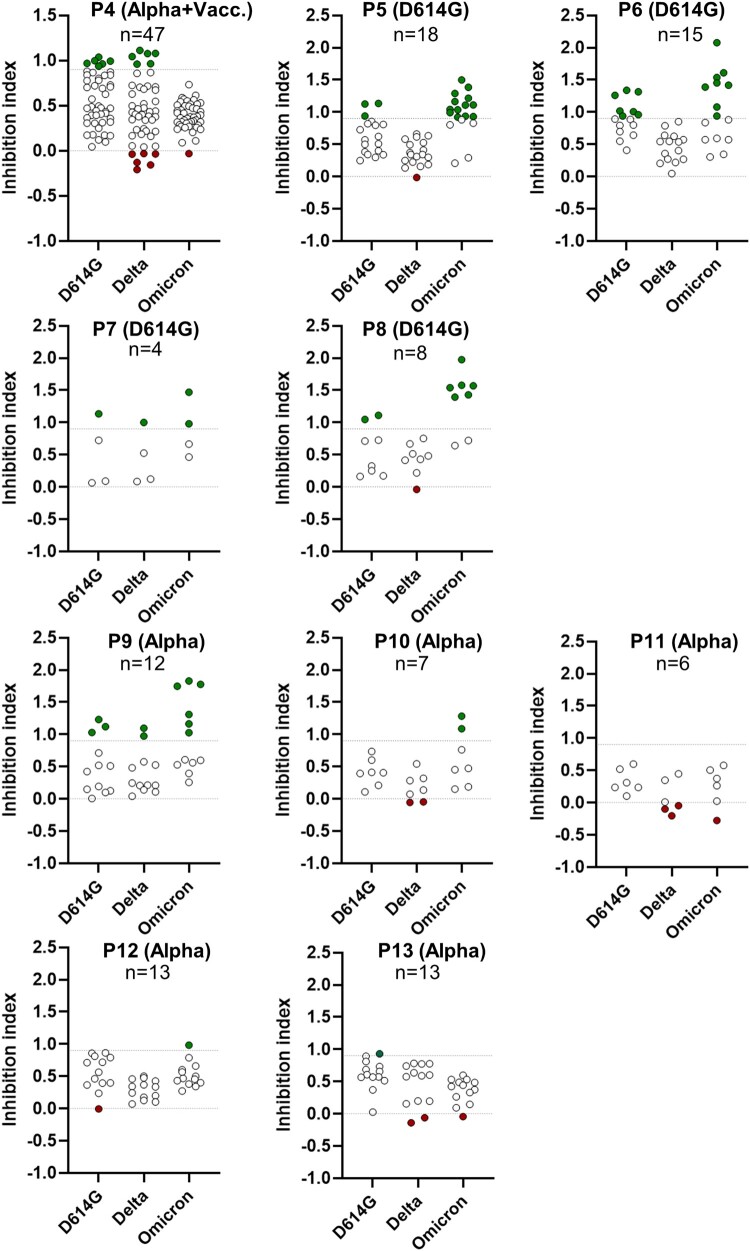
Reactivity of human monoclonal antibodies secreted by B cell clones from convalescent patients against D614G, Delta and Omicron variants. The ability of B cell clone supernatants from convalescent patients to inhibit the cytopathic effect induced by the SARS-CoV-2 variants was quantified by assessing cell viability of VeroE6/TMPRSS2 cells three days after cell infection. Green-filled symbols: Potent neutralizing Abs (inhibition index >0.9); white-filled symbols: poorly/non-neutralizing Abs (inhibition index between 0 and 0.9); red-filled symbols: enhancing Abs (inhibition index <0).

Out of the 445 mAbs subjected to testing, 98 mAbs exhibited strong neutralization activity against at least one of the three variants, while 309 mAbs were found to have no significant impact or only minor effects on the infectivity of the tested variants. Additionally, 38 mAbs were identified as enhancers of the cytopathic effects induced by SARS-CoV-2 (Figure 1(a)). The functional neutralizing and enhancing properties of representative mAbs were validated using three different approaches from the standardized screening assay (Supplementary Fig. 1). The percentages of neutralizing and infectivity-enhancing Abs varied significantly among donors, ranging from 0% to 100% for neutralizing mAbs and from 0 to 50% for enhancing mAbs (Figure 1(b–d), Figure 2). Interestingly, certain convalescent patients, such as Patient P1, exhibited a combination of both neutralizing and enhancing Ab clones (Figure 1(b)). In contrast, some donors displayed a substantial preponderance of neutralizing Ab clones (Figure 1(c)), while others exhibited an abundance of enhancing Ab clones (Figure 1(d)). To assess the potential impact of variable intervals from symptom onset to sampling on the B cell antibody response, we conducted a linear regression analysis for neutralizing and facilitating antibodies, excluding two vaccinated individuals with extended sampling intervals. Non-significant correlations were observed for neutralizing antibodies (slope: −0.05870, *p*-value: 0.7681, R squared: 0.01016) and facilitating antibodies (slope: −0.03785, *p*-value: 0.7122, R squared: 0.01585), suggesting no significant association between the duration from symptom onset to sampling and the frequencies of these antibodies. Collectively, these findings underscore the functional diversity and the cross-reactivity of anti-SARS-CoV-2 Abs produced following a primary infection with the SARS-CoV-2 at the monoclonal level.

### Cross-reactivity of neutralizing and enhancing monoclonal antibodies induced by SARS-CoV-2 infection

**Figure 3.**
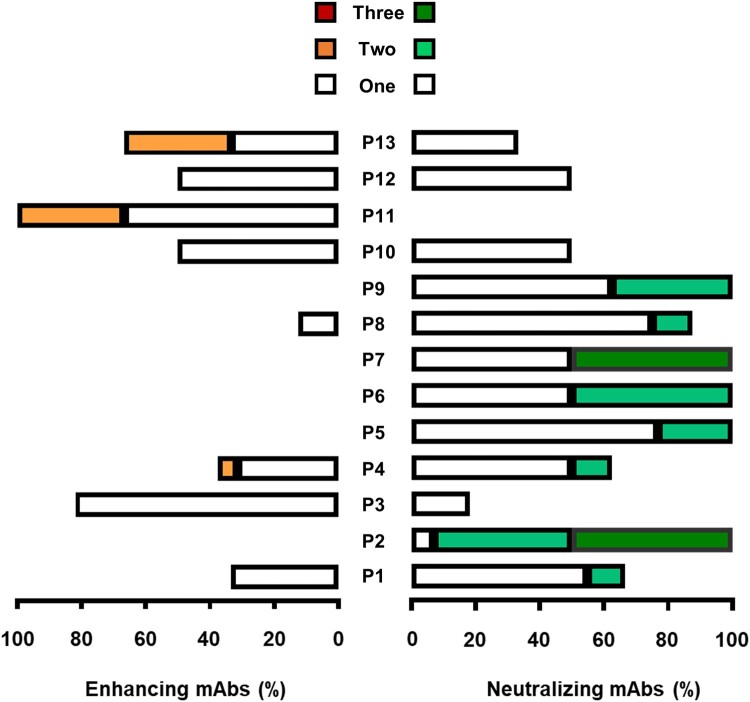
Proportion of neutralizing and facilitating antibodies per patient and cross-reactivity with D614G, Delta and Omicron variants. Lineage specific and cross-reactive anti-SARS-CoV-2 monoclonal Abs were categorized based on their ability to impede the cytopathic effect induced by the SARS-CoV-2 variants D614G, Delta and Omicron. One: reactivity towards one variant, Two: reactivity towards two variants and Three: reactivity towards three variants.

To understand how the balance between neutralizing and enhancing Abs evolves with respect to variants, we subsequently analyzed the cross-reactivity of the generated mAbs against the D614G, Delta, and Omicron variants. (Figure 3). Significant variability was observed among individuals, with some having exclusively neutralizing Abs (P2, P5, P6, P7, and P9) or only facilitating Abs (P11). In contrast, others exhibited both types of Abs in varying proportions (Figure 3). Among the 98 neutralizing Abs, 66% (*n* = 65) were capable of specifically neutralizing one variant, while 26% (*n* = 25) neutralized 2 variants, and 10% (*n* = 8) demonstrated neutralization against all three variants, namely D614G, Delta and Omicron (Figure 3).

**Figure 4.**
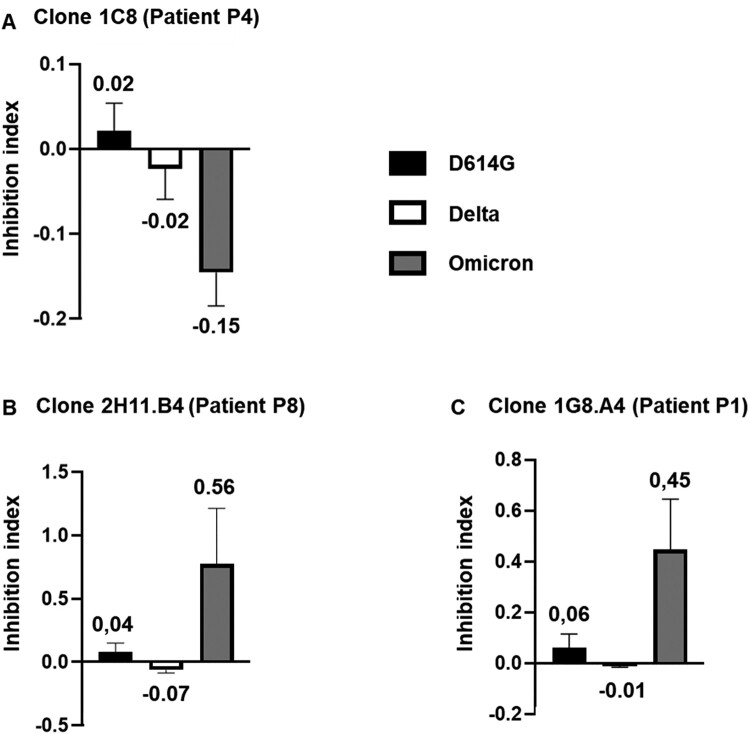
Divergent functionalities of cross-reactive monoclonal antibodies against distinct SARS-CoV-2 variants. (A) Functional properties of representative clone 1C8 generated from B cells of patient P4, exclusively facilitating the Delta and Omicron variants. The impact on cytopathic outcomes induced by D614G, Delta and Omicron variants was assessed by measuring the viability of VeroE6 cells after 3 days of incubation. The Inhibition Index was calculated by dividing the normalized cell viability obtained with clone supernatants by the normalized cell viability of the viral inhibition control (remdesivir). Data are represented as mean ± SEM of triplicates. (B,C) Identification of two cross-reactive monoclonal antibodies exhibiting neutralizing properties against Omicron and enhancing properties against Delta. The clones 1G8.A4 and 2H11.B4 were generated from B cells of patients P1 and P8, demonstrating both neutralizing effects against Omicron and enhancing effects against Delta variants. The inhibition index was calculated as described above.

Twenty-three, 15 and 27 neutralizing mAbs exhibited distinct reactivity profiles specifically directed against respectively D614G, Delta and Omicron (Table 2). Fifteen neutralizing Abs strongly neutralized both D614G and Delta, while 10 mAbs neutralized both D614G and Omicron. Surprisingly, among the 98 mAbs capable of neutralizing SARS-CoV-2, none exhibited strong neutralizing properties exclusive to Delta and Omicron. Finally, eight mAbs effectively neutralized all three variants D614G, Delta and Omicron (Table 2). Most of the identified enhancing Abs identified (90%, *n* = 35/39) increased the cytopathic effect of a specific variant, while 8% (*n* = 3/39) of mAbs elicited an Ab-dependent enhancement effect on two distinct variants (Figure 3). Specifically, five mAbs enhance the infectivity of the D614G variant, whereas 24 and 6 mAbs strongly enhanced the infectivity of Delta and Omicron variants, respectively (Table 3). Surprisingly, in contrast to what was observed with neutralizing mAbs, no mAbs enhanced both D614G and Delta, nor D614G and Omicron. However, three mAbs enhanced exclusively Delta and Omicron (Table 3, Figure 4(a)). Notably, two mAbs that increased the cytopathic effect of the Delta variant were also capable of strongly neutralizing Omicron (Figure 4(b, c)). This implies that none of the facilitating mAbs showed reactivity against the 3 variants tested, unlike the neutralizing Abs (Figure 3). Taken together, these results underscore the delicate balance between neutralizing and enhancing Abs observed at the monoclonal level in convalescent individuals. They also highlight the functional duality of certain cross-reactive Abs, potentially enabling variants to evade immunity established after a primary infection.

**Table 2.** Reactivities against SARS-CoV-2 variants of neutralizing monoclonal antibodies isolated from B cell clones of patients.

		One reactivity			Two reactivites			Three reactivi-ties
	% of neutralizing clones	D614G	Delta	Omicron	D614G/Delta	D614G/Omicron	Delta/Omicron	D614G/Delta/Omicron
**P1**	11 (28/252)	11	10	2	5	0	0	0
**P2**	100 (14/14)	1	0	0	6	0	0	7
**P3**	5 (2/36)	1	1	0	0	0	0	0
**P4**	21 (10/47)	4	4	0	2	0	0	0
**P5**	72 (13/18)	0	0	10	0	3	0	0
**P6**	67 (10/15)	2	0	3	0	5	0	0
**P7**	50 (2/4)	0	0	1	0	0	0	1
**P8**	87 (7/8)	1	0	5	0	1	0	0
**P9**	67 (8/12)	0	0	5	2	1	0	0
**P10**	29 (2/7)	2	0	0	0	0	0	0
**P11**	0 (0/6)	0	0	0	0	0	0	0
**P12**	8 (1/13)	0	0	1	0	0	0	0
**P13**	8 (1/13)	1	0	0	0	0	0	0
**Total**	** **	**23**	**15**	**27**	**15**	**10**	**0**	**8**

**Table 3.** Reactivities against SARS-CoV-2 variants of enhancing monoclonal antibodies isolated from B cell clones of patients.

		One reactivity			Two reactivites			Three reactivi-ties
	% of enhancing clones	D614G	Delta	Omicron	D614G/Delta	D614G/Omicron	Delta/Omicron	D614G/Delta/Omicron
**P1**	6 (14/252)	4	8	2	0	0	0	0
**P2**	0 (0/14)	0	0	0	0	0	0	0
**P3**	25 (9/36)	0	5	4	0	0	0	0
**P4**	13 (6/47)	0	5	0	0	0	1	0
**P5**	0 (0/18)	0	0	0	0	0	0	0
**P6**	0 (0/15)	0	0	0	0	0	0	0
**P7**	0 (0/4)	0	0	0	0	0	0	0
**P8**	13 (1/8)	0	1	0	0	0	0	0
**P9**	0 (0/12)	0	0	0	0	0	0	0
**P10**	29 (2/7)	0	2	0	0	0	0	0
**P11**	50 (3/6)	0	2	0	0	0	1	0
**P12**	8 (1/13)	1	0	0	0	0	0	0
**P13**	15 (2/13)	0	1	0	0	0	1	0
** **	** **	**5**	**24**	**6**	**0**	**0**	**3**	**0**

## Discussion

We previously demonstrated that the antiviral B cell repertoire differs significantly between convalescent patients according to the severity of the infection [15]. Here, we expanded on this notion by unveiling, at the monoclonal level, the functional diversity of Abs elicited during a primary infection with SARS-CoV-2 variants.

While the functional spectrum of antiviral B cells differed from patient to patient, most of the convalescent patients generated anti-SARS-CoV-2 Abs with limited, or even non-neutralizing capabilities against the infecting variant. The role of non-neutralizing Abs in modulating SARS-CoV-2 infection is still not well understood, but recent evidence suggests their active involvement in conferring protection against infection through viral phagocytosis [19–21]. Alongside these non-neutralizing Abs, other B cell clones secreted Abs with potent neutralizing properties against the infecting variant. As expected, and as illustrated at a monoclonal level, the initial infection with the D614G or Alpha variant resulted in a functional equilibrium between neutralizing and enhancing Abs targeting the infecting variant. Surprisingly, a notably high frequency of B cell clones producing neutralizing Abs was observed in samples from elderly patients who had experienced severe forms of COVID-19 (patients P2, P5 and P9). Strikingly, no B cell clones secreting enhancing Abs were detected in the samples from these patients. One plausible hypothesis is that the majority of these elderly patients, who endured severe manifestations of COVID-19 requiring hospitalization but successfully overcame the infection, might have developed an adaptive anti-SARS-CoV-2 response, leading to the preferential development of neutralizing antibodies. Furthermore, the absence of facilitating Abs in elderly patients with severe forms of the disease is consistent with existing literature, which establishes no correlation between disease severity and the presence of enhancing Abs [22].

Our approach also unveiled a complex interplay initiated by the primary infection, involving both lineage-specific and cross-reactive mAbs with opposing effects on the infectivity of the Delta and Omicron variants. Interestingly, we observed that among the neutralizing Abs with cross-reactivity limited to two of the three variants tested, none specifically neutralized the Delta and Omicron variants. Conversely, cross-reactive Abs with infectivity-enhancing properties were exclusively specific to the Delta and Omicron variants. It is still unknown to what extent the widespread presence of these Abs in the human population, following the initial spread of the first variants, may have influenced the selection of late variants. Nevertheless, this question seems to us of very great general importance, as it highlights a potential new mechanism for the evolution and selection of emerging pathogens.

An important finding of this study is the characterization of cross-reactive mAbs exhibiting both neutralizing and enhancing properties depending on the viral variants. Similar Abs have been previously documented in the context of dengue virus infection. In secondary dengue virus infection, when an individual is exposed to a virus of the same serotype, pre-existing Abs can effectively neutralize the virus. However, if the individual encounters a virus of a different serotype, these Abs not only lose their neutralizing ability but may even facilitate viral entry [23]. Abs with variant-dependent functions (neutralization or enhancement) have also been observed in the context of SARS-CoV-2 infection [6,7,24,25]. Unlike dengue virus infection, the ability of these facilitating Abs to increase the severity of infection in human has not been demonstrated, and *in vivo* experiments suggest that they could retain a protective effect [7]. However, these facilitating Abs could also play a critical role in the emergence of new variants. Further investigations are required to address this question.

The mechanisms responsible for the phenomenon of Ab-dependent enhancement, as reported in this study, are not yet fully understood. Generally, Ab-dependent enhancement is thought to occur through the binding of infectious virus-Ab complexes to Fcγ receptors found on myeloid cells, leading to virus internalization[26,27]. In contrast, the mechanism by which the facilitating Abs described here operate does not rely on Fcγ receptor expression, as VeroE6 cells do not express this receptor. A similar enhancement mechanism of infection in Fcγ receptor-negative cells has been previously documented with tick-borne encephalitis virus, West Nile virus, and Sindbis virus [28–30]. In the context of SARS-CoV-2, the phenomenon of *in vitro* enhancement by Abs independent of Fcγ receptors has been also documented [7,31]. These enhancing Abs specifically target epitopes within the NTD of the spike protein. While the precise mechanisms underlying Ab-mediated enhancement in Fcγ receptor-negative cells are still under investigation, it appears to involve Ab-induced conformational changes that enhance the binding of virions to the cell surface. This process prompts the RBD to adopt an open conformation, increasing its affinity for ACE2 and consequently amplifying infectivity. It is hypothesized that each infectivity-enhancing Ab binds to a particular site on the NTD of two adjacent trimeric spike proteins, inducing motion in the spike protein [32]. This implies that the RBD adopts an open conformation, rendering it highly infectious. The extent to which the identified facilitating Abs operate through a similar mechanism is currently under scrutiny. However, among the 38 enhancing Abs identified, only 22 target the spike protein, hinting at the existence of other facilitation mechanisms independent of Fcγ receptors.

Another important consideration is the implication of our findings regarding the current variants of SARS-CoV-2, including variants like BQ.1.1, XBB, and more recently, JN.1 (an offspring of BA.2.86). Although our study did not specifically assess antibody reactivity against these newer variants, our methodology focused on strains that exhibited significant antigenic distance from the B.1 lineage and had substantial impact on viral transmission. Considering the ongoing evolution of variants, individual functionalities of B cell clones may undergo changes in response to these variants. However, the fundamental balance we have highlighted between neutralizing and enhancing antibodies within the B cell repertoire of infected individuals will persist alongside the virus's antigenic evolution and the adaptive nature of humoral responses.

Our findings carry significant implications for safety considerations, strongly advocating for the assessment of Ab-dependent enhancement phenomenon against each emerging new variants when considering neutralizing mAbs for Abs-based therapies. This becomes especially critical since November 2021, following the emergence of the Omicron variant, which has been proposed to represent the initial strain of a new SARS-CoV-2 serotype [33]. Given the wide range of cross-reactive Abs induced by specific variants, it is also crucial to evaluate the potential autoreactivity of mAbs designed for therapeutic purposes.

In summary, our data shed light on the functional diversity of cross-reactive Abs generated during a primary SARS-CoV-2 infection. The specific balance of functionality between lineage specific and cross-reactive mAbs, elicited following a primary SARS-CoV-2 infection varies at the individual level due to host-specific factors. At the population level, this diversity exerts immune-mediated selection pressure, contributing to the emergence of SARS-CoV-2 immune escape variants.

## Supplementary Material

Revised_Supplemental_Appendix_Coutant_et_al

Revised_Supplementary_Figure_1_Coutant_et_al

## Data sharing statement

Data are available within the article.
