# Supplementary material for: Neutralizing and enhancing monoclonal antibodies in SARS-CoV-2 convalescent patients: lessons from early variant infection and impact on shaping emerging variants
Source: Emerg Microbes Infect. 2024 Jan 19;13(1):2307510. doi: 10.1080/22221751.2024.2307510 (PMC10829827; doi:10.1080/22221751.2024.2307510)
Supplement: Revised_Supplemental_Appendix_Coutant_et_al [file TEMI_A_2307510_SM1344.docx]

**Supplementary appendix**

**Supplemental to: Neutralizing and Enhancing Monoclonal Antibodies in SARS-CoV-2 Convalescent Patients: Lessons from Early Variant Infection and Impact on Shaping Emerging Variants**

Supplementary Material & Methods **2**

Supplementary Figure Legends **4**

**Supplementary Material and Methods**

**Virus isolate**

SARS-CoV-2 Omicron BQ.1 (hCoV-19/France/HDF-IPP49210/2022) was isolated by the National Reference Center for Respiratory Viruses hosted by Institut Pasteur (Paris, France). The sequence is available on GISAID : EPI_ISL_14778228

**Virus quantification by indirect immunofluorescence**

Vero E6 cells were initially seeded into 96-well plates (Nunc, Roskild, Denmark) at a density of 1x10^4^ cells per well one day before the experiment. The cells were exposed to mixtures containing clone supernatants and virus and then incubated for 24 hours at 37°C. The mixtures were prepared by combining a fixed quantity of virus equivalent to 100 plaque-forming units (PFU) with an equal volume of clone supernatants (resulting in a final concentration of 1 µg/mL) and incubating them at room temperature for 30 minutes. After 24 hours, the cells were fixed with ice-cold acetone (95%) and then incubated with a pool of calibrated convalescent patient serum (1:150) for 40 minutes at 37°C. After washing, cells were incubated with FITC-conjugated anti-human IgG, IgA, and IgM antibodies (Abliance, Compiègne, France) at a 1:300 dilution for 40 minutes at 37°C. Each experimental condition was performed in duplicate, and the enumeration of PFU was independently conducted by two readers. Cell imaging was conducted using an Axioplan 2 imaging microscope (Zeiss, Munich, Germany).

**Fluorescence quantification of Spike protein expression**

Fluorescence quantification of Spike protein expression at the surface of Vero E6 TMPRSS2 cells was conducted by exposing the cells to mixtures of clone supernatants and virus particles, as previously described. After 24 hours of exposure, the fluorescence and cell confluence were quantified using the Incucyte ® S3 Live-Cell Analysis Systems (Sartorius) according to the manufacturer’s instructions, with an acquisition time of 800 ms for the green channel. Fluorescence intensity and cell confluence data from each well were obtained using the Incucyte 2020B software (Sartorius).

**Extraction and quantification of Viral RNA by qRT-PCR**

100µL of viral supernatant was collected in S-Block (Qiagen) previously loaded with VXL lysis buffer containing proteinase K and RNA carrier. RNA extraction was performed using the Qiacube HT automat and the QIAamp 96 DNA kit HT following manufacturer instructions. Cells were lysed in 140µl of Buffer RLT (Qiagen) and total intracellular RNA of each well was extracted using RNeasy 96 HT kit (Qiagen) with the Qiacube HT automat following manufacturer’s instructions. Viral RNA was quantified by real-time RT-qPCR (GoTaq 1-step qRt-PCR, Promega) using 3.8µL of extracted RNA and 6.2µL of RT-qPCR mix and standard fast cycling parameters, i.e., 10min at 50°C, 2 min at 95°C, and 40 amplification cycles (95°C for 3 sec followed by 30sec at 60°C). Quantification was provided by four 2 log serial dilutions of an appropriate T7-generated synthetic RNA standard of known quantities (102 to 108 copies/reaction). RT-qPCR reactions were performed on QuantStudio 12K Flex Real-Time PCR System (Applied Biosystems) and analyzed using QuantStudio 12K Flex Applied Biosystems software v1.2.3. Primers and probe sequences, which target SARS-CoV-2 N gene, were: Fw: GGCCGCAAATTGCACAAT; Rev: CCAATGCGCGACATTCC; Probe: FAM-CCCCCAGCGCTTCAGCGTTCT-BHQ1.

**Supplementary Figure Legends**

**Supplementary Figure 1: Validation of neutralizing and enhancing properties of monoclonal antibodies by distinct approaches.** The neutralizing properties (clone 3H1A10 from patient P4) and enhancing properties (clone 4B6.01 from patient P5) of the generated monoclonal antibodies were assessed using three distinct approaches: (A) Indirect immunofluorescence on fixed VeroE6 cells exposed to SARS-CoV-2 (Omicron BQ.1) and clone supernatants (final concentration: 1 µg/mL) for 24 hours. Images were captured at 100x magnification; (B) Dose-response effect of clones 3H1A10 and 4B6.01. (C) Quantification of Spike protein expression on the surface of VeroE6 TMPRSS2 cells after 24 hours of incubation with either medium alone (MC: medium control), virus alone (VC: virus control), or a mixture of virus and clone supernatant. Results are presented as mean ± SEM measured in 27 fields. (D) Quantification of intracellular viral RNA by qRT-PCR after 24-hour incubation of VeroE6 TMPRSS2 cells exposed to virus alone or virus mixed with clone supernatants. Results are represented as mean ± SEM of n=3 independent experiments. Two-tailed non-parametric Mann-Whitney tests were used for comparisons.* p < 0.1, *** p < 0.001, **** p<0.0001.
