# Supplementary figures and images for: Neutralizing and enhancing monoclonal antibodies in SARS-CoV-2 convalescent patients: lessons from early variant infection and impact on shaping emerging variants
Source: Emerg Microbes Infect. 2024 Jan 19;13(1):2307510. doi: 10.1080/22221751.2024.2307510 (PMC10829827; doi:10.1080/22221751.2024.2307510)

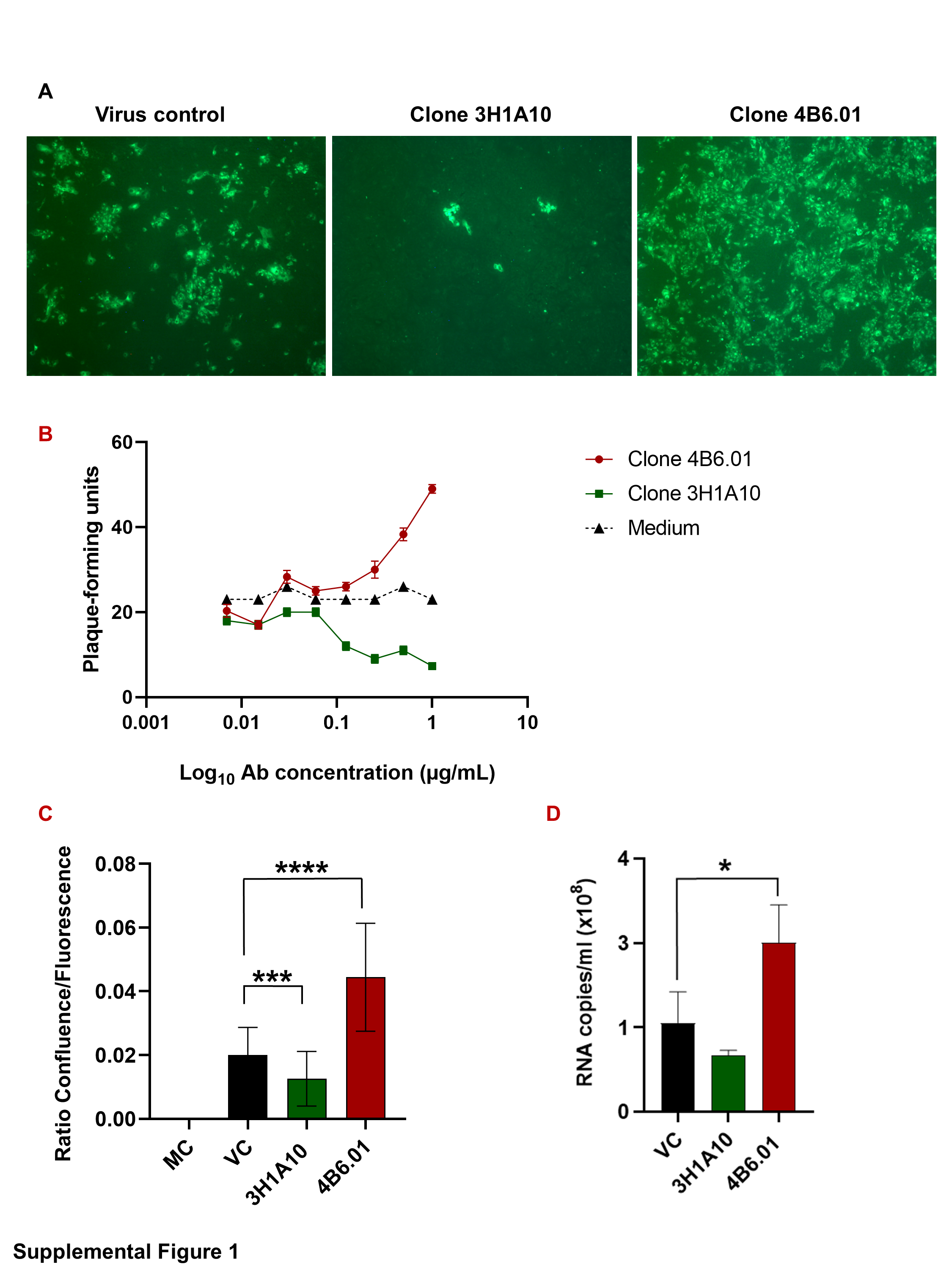

Supplement: Revised_Supplementary_Figure_1_Coutant_et_al [file TEMI_A_2307510_SM1295.tif]
